# *Culex pipiens pallens* cuticular protein CPLCG5 participates in pyrethroid resistance by forming a rigid matrix

**DOI:** 10.1186/s13071-017-2567-9

**Published:** 2018-01-04

**Authors:** Yun Huang, Qin Guo, Xiaohong Sun, Cheng Zhang, Na Xu, Yang Xu, Dan Zhou, Yan Sun, Lei Ma, Changliang Zhu, Bo Shen

**Affiliations:** 1 Department of Pathogen Biology, Nanjing Medical University, Nanjing, China; 2 Jiangsu Province Key Laboratory of Modern Pathogen Biology, Nanjing Medical University, Nanjing, China

**Keywords:** Insecticide resistance, Mosquito cuticle, Cuticular protein, RNA interference, Transmission electron microscopy

## Abstract

**Background:**

Chemical insecticides have hugely reduced the prevalence of vector-borne diseases around the world, but resistance threatens their continued effectiveness. Despite its importance, cuticle resistance is an under-studied area, and exploring the detailed molecular basis of resistance is critical for implementing suitable resistance management strategies.

**Methods:**

We performed western blotting of cuticular protein CPLCG5 in deltamethrin-susceptible (DS) and laboratory-produced deltamethrin-resistant (DR) strains of *Culex pipiens pallens.* Immunofluorescence assays using a polyclonal antibody to locate cuticular CPLCG5 in mosquitoes. EM immunohistochemical analysis of the femur segment was used to compare the cuticle in control and CPLCG5-deficient siRNA experimental groups.

**Results:**

The gene *CPLCG5* encodes a cuticle protein that plays an important role in pyrethroid resistance. Based on a prior study, we found that expression of CPLCG5 was higher in the resistant (DR) strain than the susceptible (DS) strain. *CPLCG5* transcripts were abundant in white pupae and 1-day-old adults, but expression was dramatically decreased in 3-day-old adults, then remained stable thereafter. Western blotting revealed that the CPLCG5 protein was ~2.2-fold higher in the legs of the DR strain than the DS strain. Immunofluorescence assays revealed CPLCG5 expression in the head, thorax, abdomen, wing, and leg, and expression most abundant in the leg and wing. EM immunohistochemical analysis suggested that the exocuticle thickness of the femur was significantly thinner in the CPLCG5-deficient siCPLCG5 strain (0.717 ± 0.110 μm) than the siNC strain (0.946 ± 0.126 μm). Depletion of CPLCG5 by RNA interference resulted in unorganised laminae and a thinner cuticle.

**Conclusions:**

The results suggest CPLCG5 participates in pyrethroid resistance by forming a rigid matrix and increasing the thickness of the cuticle.

**Electronic supplementary material:**

The online version of this article (doi: 10.1186/s13071-017-2567-9) contains supplementary material, which is available to authorized users.

## Background

Over the past decade, the insecticide pyrethroid has been used extensively and continuously to control mosquito insect vectors. Mosquito control by insecticides is a core component of mosquito-borne disease control programs [1], and pyrethroid has also been used to control other pests [2, 3]. According to World Health Organisation (WHO) data from 2010, widespread pyrethroid resistance increased between 2000 and 2010 [4], and such resistance threatens the effectiveness of global mosquito-borne disease control efforts [5–7]. Mosquito-borne diseases such as malaria, dengue fever, and Zika fever, constitute a major burden on public health worldwide [8–12]. A thorough understanding of insecticide metabolism may help us to generate countermeasures to insecticide resistance that may be critical for effective mosquito control.

In general, three types of resistance mechanisms have been described [13–15]: cuticular resistance, metabolic resistance, and target-site resistance [16]. In 1963, an investigation conducted on house flies to determine the causes of insecticide resistance revealed that penetration in the resistant (DR) strain was slower than in the sensitive (DS) strain, indicating resistance to both DDT and pyrethroids [17]. A study showed that pyrethroid-resistant *Anopheles funestus* females were more likely to have thicker cuticles than susceptible females, and females generally had thicker cuticles than males [18]. Furthermore, Lilly et al. [19] demonstrated that cuticle thickening was present within a pyrethroid-resistant strain of the bed bug *Cimex lectularius*. Cuticle analysis by electron microscopy and characterisation of lipid extracts indicated that resistant mosquitoes had a thicker epicuticular layer and higher cuticular hydrocarbon (CHC) content (~29%) [20]. Multiple studies have since confirmed that cuticular thickness in the DR strain is much thicker than that in the DS strain [19, 20].

The cuticle consists of two layers, an inner procuticle and an outer epicuticle. The procuticle is comprised of the endocuticle and exocuticle. The endocuticle is composed of chitin and proteins, and proteins in the outer part are sometimes hardened (tanned) to form a dark exocuticle. The endocuticle contains two important properties, flexibility and elasticity, which support movement but only to a limited extent. The hard exocuticle of the exoskeleton provides a rigid support for the attachment of muscles, as well as a protective covering. The epicuticle is mainly responsible for water impermeability, which has enabled insects to colonise dry environments despite their small size and large surface area to volume ratio. The epicuticle must first be penetrated by insecticides before their toxic effects can be exerted [21–24], and proteins are the major constituents of insect cuticles and determine their properties.

The cuticular protein (CP) family was first discovered in cast cuticles from *Anopheles gambiae* by tandem mass spectrometry in 2007 [25]. Most gene family members have the prefix ‘CPLC’ which stands for ‘cuticular protein of low complexity’ members are particularly important in protein-protein interaction networks [26, 27]. Studies revealed that the mature TcCP30 protein has a low-complexity sequence and undergoes laccase2-mediated cross-linking during cuticle maturation [28]. Even in the human malaria parasite, the low complexity region was shown to be responsible for protein-protein interaction in the enzyme complex [29]. In *Anopheles*, transcript levels of two CPLCGs were more than two-fold higher in pyrethroid-resistant vs pyrethroid-sensitive mosquitoes, which suggests that CPLCGs participate in insecticide resistance [11]. As reported previously, mRNA levels of *CPLCG5* indicate a role for the protein in deltamethrin resistance in *Culex pipiens pallens* [30]. Thus, herein we explored the function of CPLCG5 in insecticide resistance.

## Methods

### Mosquito strains

The *Cx. pipiens pallens* strains used in this study were from Tangkou Village (Shandong Province) and have been maintained in our lab without exposure to any insecticides since 2009, hence they are susceptible to insecticides and served as the DS strain. The deltamethrin-resistant (DR) strain used in this study was generated from the DS strain by repeated selection for 84 generations at the larval stage in the presence of deltamethrin at the 50% lethal concentration (LC_50_), and was defined as the Lab-DR strain. The LC_50_ values of the DS and DR strains were 0.03 and 3.42 mg/l, respectively. The selection procedure was performed as described previously [31, 32]. Bottle assays (at 7 mg/l deltamthrin) found that the time taken to knockdown 50% of the test population (KDT50) for the DR strain was 2 h compared with 25 min for the DS strain. Mosquitoes were reared at 27−28 °C with 70–80% humidity under a 12:12 h light:dark photoperiod. All adult mosquitoes were provided with 10% (wt/vol) sucrose solution [33–35].

### Protein extraction and identification

Legs of 3-day-old adult mosquitoes of the two strains (*n* = 40) were dissected and homogenised in 300 μl of cold 0.1 M phosphate-buffered saline (PBS) pH 7.4 containing proteinase inhibitor cocktail (Cell Signaling, Danvers, USA). The homogenate was centrifuged at 13,000× *g* for 2 min at 4 °C, and the supernatant was collected as the PBS soluble fraction. The pellet was resuspended in 100 μl of SDS-PAGE sample buffer, heated at 95 °C for 10 min, centrifuged at 13,000× *g* for 2 min, and the supernatant was collected as the SDS-PAGE soluble fraction. Protein extracts were analysed by 4−20% gradient gel electrophoresis, and gels were stained by silver staining. The appropriate protein band was then selected, excised, digested with trypsin, and the resulting fragments were analysed by liquid chromatography-tandem mass spectrometry [25, 36, 37].

### Real-time PCR

Total RNA isolation, first strand cDNA synthesis, and real-time PCR were performed as described previously [30]. Total RNA was isolated from five female adults 3 days after merging for each biological replicate. Primers used for real-time PCR experiments are listed in Additional file 1: Table S1, and were synthesised by BGI (Shenzhen, China).

### RNA interference (RNAi)

The template for generating the siRNA for CPLCG5 (siCPLCG5) was made using PCR by GenePharma (Shanghai, China) with the primer set shown in Additional file 1: Table S1. siCPLCG5 (500 ng per insect) was injected into the thorax of 12 adult female DR strain mosquitoes. A scrambled siRNA (SiNC) was also synthesised and injected to serve as a negative control. To analyse the knockdown level of *CPLCG5* transcripts after RNAi, total RNA was isolated from whole insects (3-day-old adults; *n* = 5). Total RNA was independently isolated for each of the three replicates, and significant differences were analysed using the Student’s t-test. CDC bottle assays were used to compare resistance levels of the siNC and siCPLCG5 groups.

### Antibody preparation

Further validation of CPLCG5 was conducted by western blotting. Rabbit polyclonal antibodies were prepared against CPLCG5. Anti-peptide antibody interaction with unique regions of CPLCG5 helps to maintain a high degree of binding specificity. Therefore, we designed a peptide for CPLCG5 based on the amino sequence of the *Cx. pipiens pallens* protein (NCBI ID: V9ZA37_CULPA). The peptide sequence shown in Additional file 2: Table S2 was also confirmed in *Cx. pipiens pallens* by PCR. Peptide synthesis and polyclonal antibody production were implemented by ABGENT (Suzhou, China) [37, 38]. Although the polyclonal antibody also detected the similar cuticular protein CPLCG3, which is difficult to distinguish from CPLCG5, LC-MS/MS analysis of the excised gel band detected only CPLCG5.

### Western blotting

Western blotting analysis was performed to evaluate the specificity of the anti-CPLCG5 antiserum and to compare the CPLCG5 expression between DR and DS strain. Legs were dissected from 3-day-old adults (*n* = 40), homogenised in 300 μl PBS containing protease inhibitor cocktail (Cell Signaling), and centrifuged at 13,000× *g* for 2 min at 4 °C. The pellet was dissolved in 100 μl of SDS sample buffer, heated at 95 °C for 10 min, centrifuged at 13,000× *g* for 2 min, and the supernatant was collected for further western blotting analysis. Protein samples were analysed by 15% SDS-PAGE followed by western blotting using the anti-CPLCG5 polyclonal antibody. Western blotting was performed as described previously [38].

### CDC bottle assay

A modified protocol for CDC bottle bioassays was used [39]. Prior to performing assays, 250 ml glass bottles were coated the previous evening with 7 mg/l deltamethrin dissolved in acetone and used as experimental group bottles, while control bottles were impregnated with acetone only. Approximately 25 3-day-old non-blood-fed female mosquitoes from the DEPC microinjection, siNC, and siCPLCG5 groups were introduced into bottles and observed for knockdown for 120 min. Three replicates were performed for each group.

### Immunofluorescence analysis

Paraffin sections were made from 3-day-old DR strain mosquitoes at the 12 h adult stage of development. Tissues were rinsed with PBST (0.01 M PBS pH 7.4 containing 0.1% Tween 20) three times for 5 min each time to remove the tissue compound, then blocked with blocking buffer (2% bovine serum albumin in PBST) for 1 h at room temperature. Sections were incubated with anti-CPLCG5 antibody (1:100 in 2% BSA in PBST) at 4 °C overnight. After washing sections with PBST three times for 5 min each time, fluorescence anti-rabbit IgG (Cell Signaling) secondary antibody was added and incubated for 50 min at room temperature. Sections were washed with PBST three times for 5 min each time at room temperature, nuclei were stained with DAPI for 10 min in the dark, and visualised and imaged using a fluorescence microscope.

### Transmission electron microscopy

Mosquitoes at 3 days old that were injected with siCPLCG5 or siNC at the 12 h adult stage of development were collected and fixed in a mixture of 4% paraformaldehyde and 0.1% glutaraldehyde in 0.1 M PBS (pH 7.4) for 24 h at room temperature. Samples were rinsed three times for 15 min each time with 0.1 M PBS, then dehydrated in a progressive ethanol gradient of 50, 60, 70, 80, 90, 95 and 100% for 15 min each time. Tissues were placed in LR white resin as follows: 2:1 ethanol:resin for 4 h, 1:1 ethanol:resin for 4 h, 1:2 ethanol:resin for 4 h, and 100% resin overnight. Samples were then embedded in LR white resin and polymerised at 40 °C for 48 h followed by ultrathin sectioning, and sections (~90 nm) were stained with 4% aqueous uranyl acetate for 10 min and imaged using a transmission electron microscope (TEM) [37]. For immunogold labelling, ultrathin sections (~90 nm) were blocked with 0.01 MPBS (pH 7.4) containing 2% BSA for 1 h, then incubated with anti-CPLCG5 antibodies (1:100) in 0.01 M PBS containing 0.02% TWEEN 20 overnight at 4 °C. Samples were rinsed with 0.01 M PBS six times for 5 min each time, followed by incubation with goat anti-rabbit IgG secondary antibody conjugated with 6 nm gold particles (1:20; Jackson) in 0.01 M TBS (pH 8.0) containing 0.02% TWEEN for 2 h at room temperature. Samples were washed with 0.05 M PBS five times for 5 min each time, then with deionised water three times for 5 min each time at room temperature, and stained with 4% aqueous uranyl acetate for 10 min.

## Results

### Identification of CPLCG5 in leg extracts from *C. pipiens pallens*

**Fig. 1:**
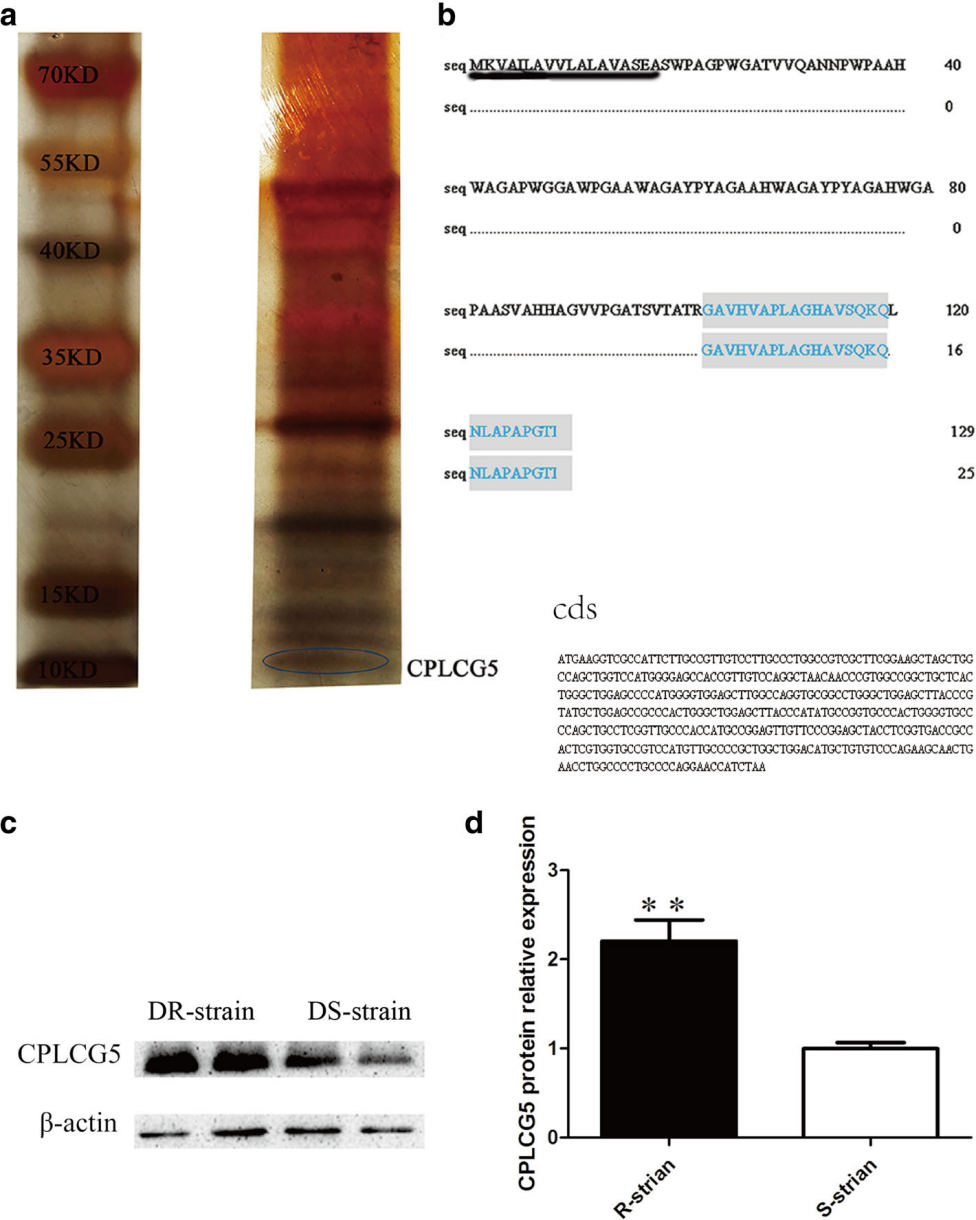
Identification of the CPLCG protein CPLCG5 in leg extracts from *Culex pipiens pallens*. **a** Analysis of cuticle protein leg extracts from 3-day-old adult mosquitoes (*n* = 40) by gradient gel electrophoresis (4−20%). The 12.7 kDa band was excised, digested with trypsin, and analysed by LC-MS/MS. **b** Nucleotide and deduced amino acid sequences of CPLCG5. The protein band was excised from the gel, digested with trypsin, and resulting peptides were analysed by LC-MS/MS. Proteins with two matched peptides are highlighted in grey, and include N103-K119 and N121-K129. N1-K18 is a predicted signal peptide. **c**, **d**, Western blotting analysis of CPLCG5 revealing ~2.2-fold higher protein levels in the DR strain than the DS strain (t-test, *t*_(4)_ = -4.917, *P* = 0.008; **P < 0.01)

As reported previously [30], CPLCG5 was significantly overexpressed in deltamethrin-resistant (DR) *C. pipiens pallens*, and expression was higher than in the DS-strain, as demonstrated by matching expression patterns (Additional file 3: Figure S1). We collected cuticle protein extracts from legs dissected from 3-day-old adult mosquitoes, and CPLCG5 isolated from these extracts was ~12.7 kDa based on its amino acid sequence. Bands on a 4−20% gradient gel (Fig. 1a) were excised, digested with trypsin, and analysed by LC-MS/MS. Comparison of with cast cuticles of *An. gambiae* by tandem mass spectrometry [25] and an online database (http://gnRetrieve/ID mapping Contact Help UniProtKB/) revealed a single candidate gene encoding the protein (V9ZA37_CULPA). The protein contains two matching peptides (highlighted in grey in Fig. 1b, Additional file 4: Figure S2). We searched the *Culex quinquefasciatus* genome, and peptides were matched to cuticular protein gene *CPIJ003477*, which shares 99% homology with V9ZA37_CULPA (originating from *Culex pipiens pallens*) with only one amino acid difference. Hence, we think these two genes are homologous. Peptide coverage for the mature CPLCG5 protein excluding the predicted signal peptide was 9.3%. Western blotting of leg extracts from 3-day-old insects (*n* = 40) revealed that expression of CPLCG5 was ~2.2-fold higher in the DR strain than the DS strain (Fig. 1c, d).

The *CPLCG5* gene encodes a protein of 129 amino acid residues containing a putative signal peptide sequence with a theoretical molecular weight of 23.7 kDa and a pI of 9.6 for the mature protein. CPLCG5 includes two invariant glycine residues in the conserved domain separated by eight amino acids, and has a conserved motif at the C-terminus [40].

### Expression of *CPLCG5* during development

**Fig. 2:**
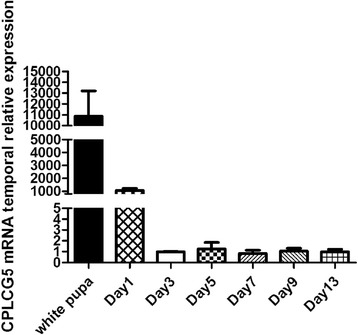
Temporal expression of CPLCG5 transcripts. Real-time PCR was performed to analyse the expression pattern of CPLCG during development. *CPLCG5* transcripts are higher in 1-day-old adults and white pupae, but decline soon thereafter up to the 3-day-old adult stage, then remain stable

Real-time PCR was performed to analyse the expression pattern of *CPLCG5* during development. *CPLCG5* transcript levels dramatically increased in white pupae (just after pupation, before they became immediately darker) and 1-day-old adults, but then declined in 3-day-old adults. *CPLCG5* mRNA levels then remained stable in 3-, 5-, 7-, 9-, and 13-day-old adults (Fig. 2). Thus, in the following functional study, we injected siCPLCG5 in 12 h adults to knock down CPLCG5 expression.

### Immunofluorescence

**Fig. 3:**
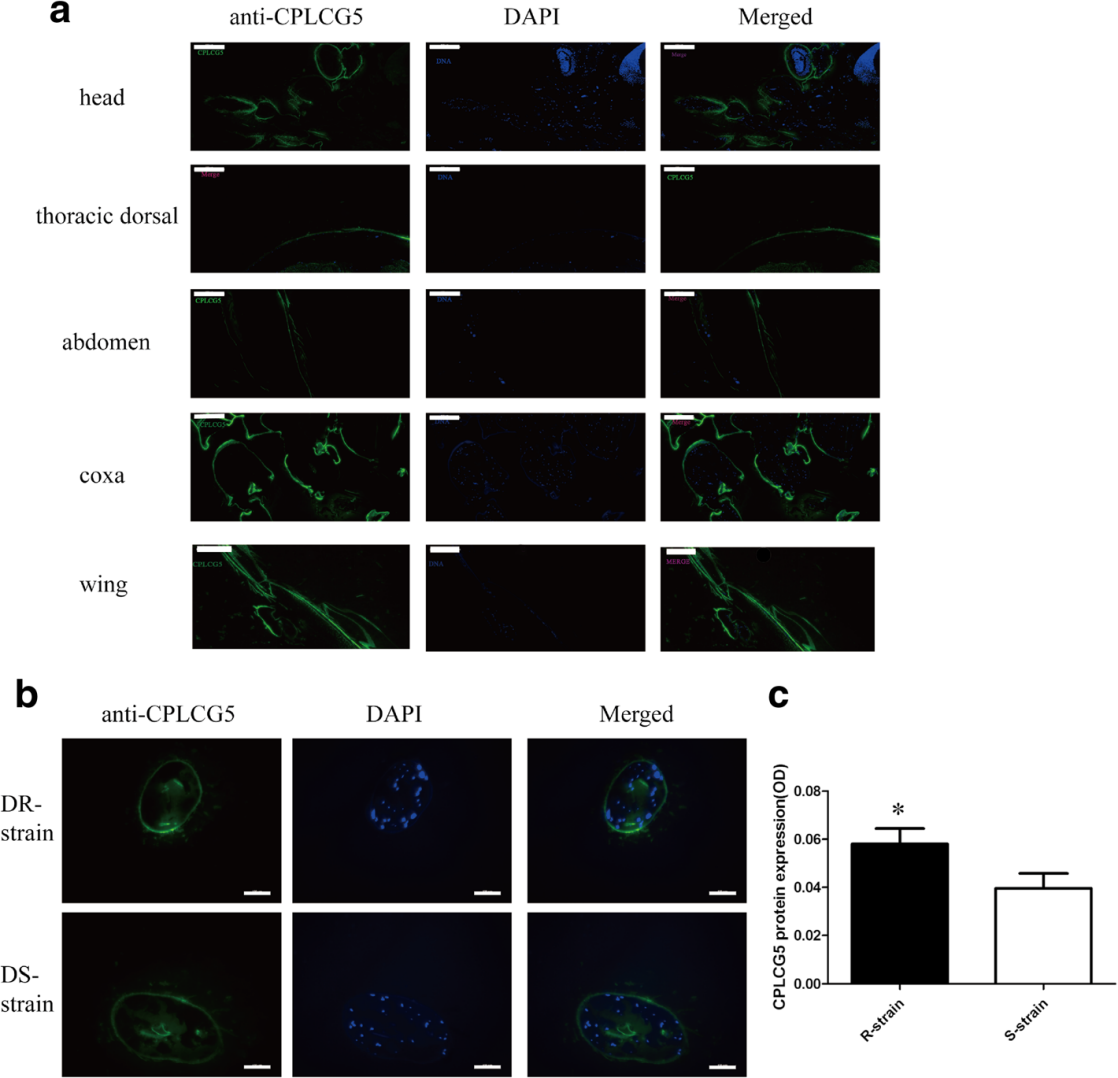
Localisation of CPLCG5 protein in adult mosquito cuticle. **a** Representative images for CPLCG5 localised in different parts of adult mosquitoes. Immunofluorescence analysis was performed to determine the location of CPLCG5 in the adult cuticle. Paraffin sections of 3-day-old adults were incubated with anti-CPLCG5 antibody, and then detected by goat anti-rabbit IgG (green). Each immunofluorescence panel is composed of three photos, with green fluorescence of CPLCG5 on the left, nuclei stained with DAPI in the middle, and merged photos on the right. The results showed that CPLCG5 protein is mainly located in the coxa. **b** Representative images of CPLCG5 localisation in the femur of DR and DS strains. Each immunofluorescence panel is composed of three photos, with green fluorescence of CPLCG5 on the left, nuclei stained with DAPI in the middle, and merged photos on the right. **c** Quantification of CPLCG5 protein expressed in the femur of DR and DS strains. Indicates a significant difference (t-test, *t*_(24)_ = 2.071, *P* = 0.0493; *P < 0.05). *Scale-bars*: **a**, 100 μm; **b**, 100 μm

In 3-day-old adults, CPLCG5 protein was detected in the cuticles of the thoracic body wall, wing, ventral abdomen and leg (Fig. 3a). Expression levels were higher in the leg and wing than the rest of the body (Fig. 3a). Although the thoracic body wall was darker and tougher in terms of consistency, CPLCG5 protein expression was low (Fig. 3a). In the subsequent experiment, we chose to study the role of CPLCG5 in the leg, and the immunofluorescence results indicated higher expression in the DR strain (Fig. 3b, c), consistent with the results of western blotting and qPCR.

### siRNA-mediated loss of function of CPLCG5

**Fig. 4:**
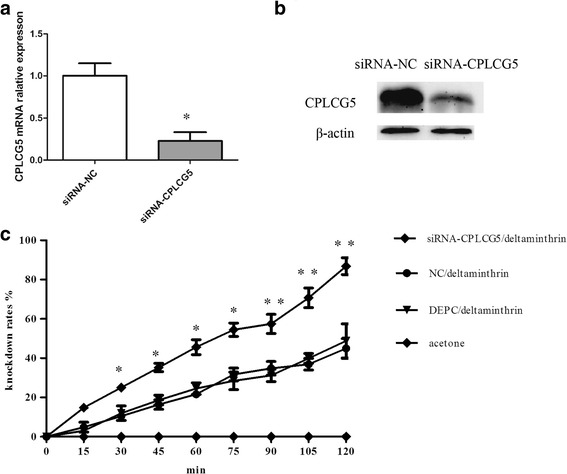
siRNA-mediated downregulation of CPLCG5. si*CPLCG5* (500 ng per insect) was injected into the thorax of 12 h female adult DR mosquitoes, and animals were recovered for 3 days. Expression of CPLCG5 was analysed by real-time PCR (**a**) and western blotting (**b**). **a** cDNAs were prepared from total RNA isolated from 3-day-old adults. The results indicate a significant difference in CPLCG5 transcript levels between control and experimental groups (t-test, *t*_(4)_ = 4.244, *P* = 0.013; *P < 0.05). Data are means ± SE. **b** Proteins were extracted from the legs of 3-day-old adults (*n* = 20) from each group. *CPLCG5* was significantly and specifically downregulated at the protein level following siRNA injection. **c** Insecticide resistance level after CPLCG5 knockdown analysed by CDC bottle assays. The results indicate that the knockdown rate of the siCPLCG5 group is higher than that of the siNC control group (Chi-square test, Additional file 5; *P < 0.05, **P < 0.01)

RNAi was used to investigate the function(s) of CPLCG5. Injection of the CPLCG5-specific siRNA led to a substantial decrease in *CPLCG5* transcripts (Fig. 4a). In addition, western blotting and qPCR showed that *CPLCG5* was strongly downregulated in the leg cuticle after siRNA injection (Fig. 4b). In this study, we demonstrated depletion of CPLCG5 at the protein level, and confirmed the high specificity of the antibody used to detect CPLCG5. For microinjection, in the siNC group, 62 mosquitoes were injected and three died after injection, equating to a mortality rate of ~5%. In the siCPLCG-5 group, 77 mosquitoes were injected and eight died after injection (mortality = 11%). Analysis of mosquito resistance by CDC bottle assays indicated that the knockdown rate in si-CPLCG5 group animals was higher than that in siNC control animals (Fig. 4c). We also performed the acetone only control for the si-CPRLCG5 group; no mosquitoes were knocked down during the 120 min observation window. Therefore, loss of CPLCG5 function could be correlated to increased susceptibility to deltamethrin, which suggests that CPLCG5 plays a critical role in insecticide resistance.

### Ultrastructure of femur segment cuticles from siCPLCG5 and siNC (control) insects

**Fig. 5:**
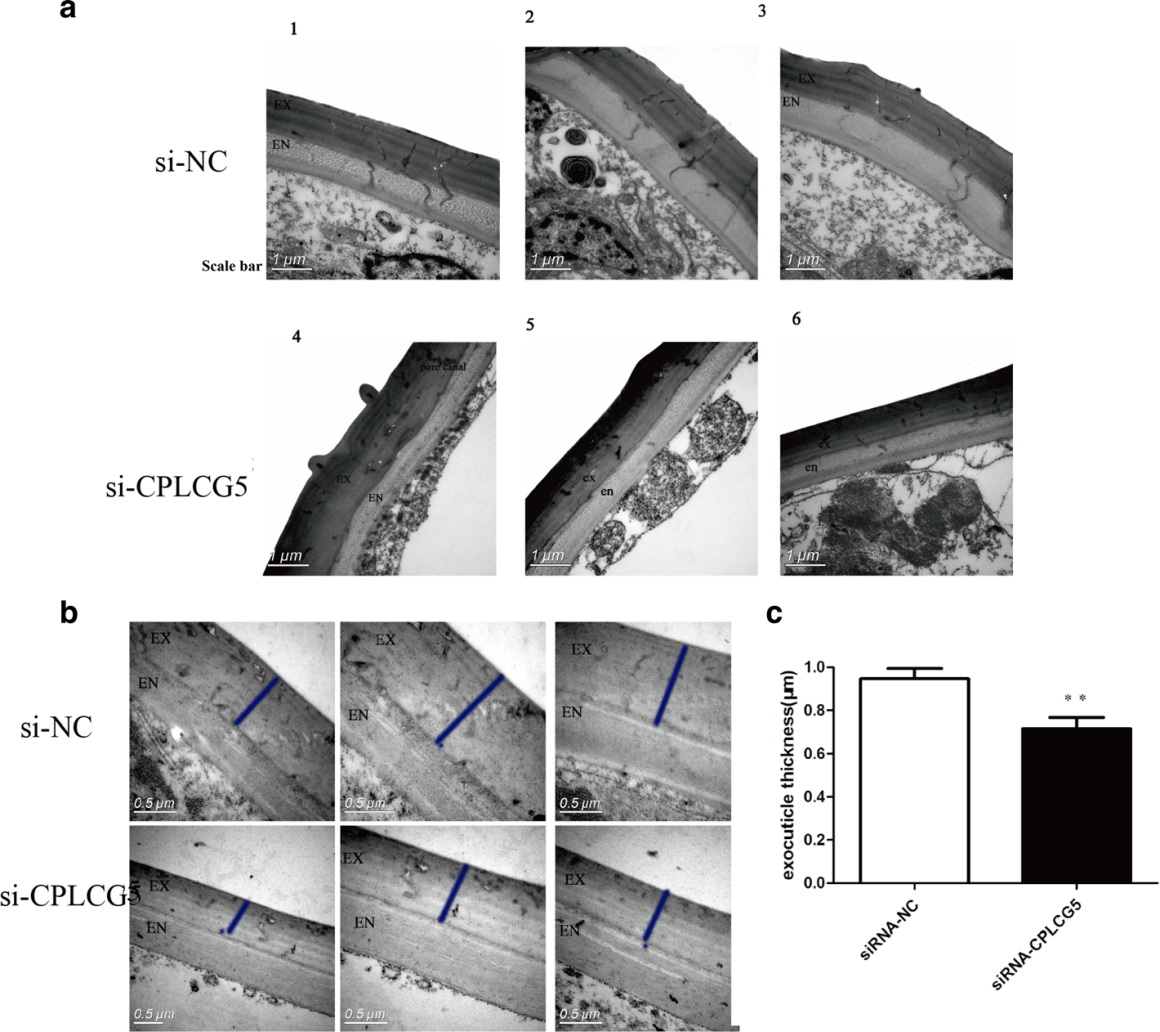
Transmission electron microscopy (TEM) analysis of the ultrastructure of mosquito femur cuticles. **a** TEM image of the mosquito femur cuticle. Adults at the 3-day-old stage were injected with si*CPLCG5* (500 ng per insect) or si*NC* (controls). The thickness of the cuticle of si*CPLCG5*-injected animals was thinner than that of the si*NC* group. **b** Gold labelling TEM of the mosquito femur cuticle. Ultrathin sections (~90 nm) were incubated with gold-labelled antibody (6 nm), and fewer gold particles were observed in si*CPLCG5*-injected animals than in si*NC*-injected animals. The blue bars indicate the exocuticle. **c** Column bar graph (vertical) of the exocuticle thickness. The graph shows the mean and SD for si*NC* (*n* = 6) and si*CPLCG5* (*n* = 5) mosquito femurs. The thickness of the exocuticle of si*NC* group animals is 0.946 ± 0.126 μm, compared with 0.717 ± 0.110 μm for si*CPLCG5* group (t-test, *t*_(10)_ = 3.262, *P* = 0.009; **P < 0.01). *Abbreviations*: EN, endocuticle; EX, exocuticle. *Scale-bars*: **a**, 1 μm; **b**, 0.5 μm

Our results demonstrated that the envelope, epicuticle and procuticle consist of numerous horizontally oriented chitinous laminae parallel to the epidermal cell apical plasma membrane in the leg cuticle of 3-day-old adult *C. pipiens pallens* (Fig. 5a). TEM analysis of leg segment cuticles from siCPLCG5-treated and siNC control insects suggested that knockdown of CPLCG5 resulted in the leg structures and chitinous parallel laminae became indistinct (Fig. 5a), whereas the pore canal became larger (Fig. 5a).

EM immunocytochemistry analysis of femur segment cuticles showed that CPLCG5 was present throughout the cuticle in 3-day-old adult insects, primarily in both the exocuticle and endocuticle (Fig. 5b). EM immunocytochemistry analysis of femur segment cuticles revealed that colloidal particles were more abundant in siNC control insects than in siCPLCG5-treated insects (Fig. 5b), suggesting that CPLCG5 was successfully knocked down in siCPLCG5-treated insects. Colloidal particles in the cuticle of siNC mosquitoes were mainly present in the exocuticle, endocuticle and the endocuticle near the epidermis. By contrast, colloidal particles in the cuticle of siCPLCG5 mosquitoes were mainly present in the exocuticle and endocuticle near the epidermis (Fig. 5b). The thickness of the femur exocuticle was significantly reduced in siCPLCG5 group (0.717 ± 0.110 μm) than in siNC group (0.946 ± 0.126 μm; *P* < 0.001; Fig. 5c).

## Discussion

Over recent decades, most researchers investigating the mechanisms of insecticide resistance have focussed their attention on metabolic resistance and target site resistance [41–43]. Our present study suggests that cuticular protein CPLCG5 is functionally involved in insecticide resistance, indicating that cuticular resistance might play an important role in the development of resistance. Cuticle thickening has been associated with pyrethroid resistance in *A. funestus* and *Triatoma infestans*, the vector carrying Chagas disease [18, 44]. Thickening of cuticle lowers penetration of insecticides, which decreases insecticide uptake and allows mosquitoes to tolerate higher concentrations of insecticides [44, 45].

Sclerotisation of the cuticle is a primary consequence of the formation of cross-links between cuticle proteins, resulting in a rigid matrix in which chitin microfibrils can be embedded. Additionally, it has been demonstrated that cuticular proteins with low-complexity sequences play an integral role as cross-linked structural proteins in the formation of lightweight rigid cuticles [46]. The CPLCG family was first identified in *A. gambiae* by proteomic analysis of cuticle preparations, and members of the CPLCG family contain low-complexity regions (LCRs) [25]. A previous study found that CPLCG proteins were most highly expressed in the endocuticle in *A. gambiae* [22]. Our present results showed that siCPLCG5 knockdown resulted in the disappearance of CPLCG5 from the endocuticle, which indicates that CPLCG5 is secreted from the epidermis, before being transported through the endocuticle to the exocuticle, where it cross-links with structural proteins to form the rigid matrix of the cuticle, resulting in thickening and toughening.

The insect cuticle is composed of several morphologically and functionally distinct layers including a waterproof envelope, an epicuticle, a procuticle and underlying epidermal cells which are responsible for producing the extracellular matrix. Using an electron microscope, we examined the cuticle structure of the *C. pipiens pallens* mosquito, and found that the endocuticle is comprised of discrete chitin fibrils orientated in a staggered fashion. Similar chitinous laminae layers have been observed in the cuticle of mosquito legs and the eggs of *Tribolium* beetles [47]. In general, cuticle differentiation is divided into three phases: establishment of layers, cuticle thickening, and orienting of chitin laminae into the typical configuration [48]. CPLCG5 underwent a steady increase in expression during the white pupal stage, but expression had decreased by 24 h after emergence. Loss of CPLCG5 function resulted in the formation of an indistinct chitinous laminae layer, larger pore canals, and a thinner cuticle, which suggests that cuticle proteins are involved in most of the cuticle differentiation phases.

*CPF3*, *CPLCG3* and *CPLCG4* mRNA transcripts are mainly located in appendages and the genitalia [22]. Similarly, in the present study, *CPLCG5* mRNAs were mainly located in mosquito appendages (legs and wings). Since both of these appendages are associated with motion, CPLCG5 may be related to flight. Additionally, CPLCG5 was expressed more highly in the legs of the DR strain, suggesting it may help these animals to avoid pesticide-treated areas, but this hypothesis requires further exploration.

## Conclusions

In this study, we investigated the role of CPLCG5 in *C. pipiens pallens*, and found that expression is highest in the legs. CPLCG5 was found to be primarily located in the cuticular ultrastructure of the procuticle. Unlike the body cuticle of this species, the femur cuticle is highly sclerotised but not highly pigmented, and remains relatively flexible. The femur cuticle consists of numerous horizontally oriented chitinous laminae lying parallel to the epidermal cell apical plasma membrane, and CPLCG5 is expressed in these structures. Depletion of CPLCG5 protein by RNAi resulted in a disorganised laminar architecture and decreased cuticle thickness, which in turn increased the susceptibility to insecticides. These results suggest CPLCG5 is critical to the formation of the lamina in the rigid and resilient cuticle of *C. pipiens pallens*, and indicate a significant role in pyrethroid resistance. The observed increase in CPLCG5 expression in DR mosquitoes correspond with thickening of the cuticle, indicative of synthetic synergy that may help to degrade cuticle proteins to improve the efficacy of insecticide penetration in diseases vectors.

## Additional files

Additional file 1: Table S1. List of primers used for qRT-PCR. (DOC 15 kb)

Additional file 2: Table S2. List of siRNA sequences used for RNA interference. (DOC 20 kb)

Additional file 3: Figure S1. qRT-PCR comparison of the expression of *CPLCG5* in DS and DR *C. pipiens pallens* strains. (PNG 39 kb)

Additional file 4: Figure S2. Representative LC-MS/MS spectrum showing a peptide from the CPLCG5 protein. (PNG 184 kb)

Additional file 5: Chi-square tests. (DOCX 19 kb)

## Abbreviations

DR: Laboratory-produced deltamethrin-resistant
DS: Deltamethrin-susceptible
LC-MS/MS: Liquid chromatography-tandem mass spectrometry
TEM: Transmission electron microscope

## References

[1] Ranson H, Lissenden N (2016). Insecticide resistance in African *Anopheles* mosquitoes: a worsening situation that needs urgent action to maintain malaria control. Trends Parasitol.

[2] Ilias A, Vassiliou VA, Vontas J, Tsagkarakou A (2017). Molecular diagnostics for detecting pyrethroid and abamectin resistance mutations in *Tetranychus urticae*. Pestic Biochem Physiol.

[3] Kasai S, Sun H, Scott JG (2016). Diversity of knockdown resistance alleles in a single house fly population facilitates adaptation to pyrethroid insecticides. Insect Mol Biol.

[4] Butler D (2011). Mosquitoes score in chemical war. Nature.

[5] Hemingway J, Field L, Vontas J (2002). An overview of insecticide resistance. Science.

[6] van den Berg H, Zaim M, Yadav RS, Soares A, Ameneshewa B, Mnzava A (2012). Global trends in the use of insecticides to control vector-borne diseases. Environ Health Perspect.

[7] Rivero A, Vézilier J, Weill M, Read AF, Gandon S (2010). Insecticide control of vector-borne diseases: when is insecticide resistance a problem?. PLoS Pathog.

[8] Garcez PP, Nascimento JM, de Vasconcelos JM, Madeiro da Costa R, Delvecchio R, Trindade P (2017). Zika virus disrupts molecular fingerprinting of human neurospheres. Sci Rep.

[9] Chareonviriyaphap T, Bangs MJ, Suwonkerd W, Kongmee M, Corbel V, Ngoen-Klan R (2013). Review of insecticide resistance and behavioral avoidance of vectors of human diseases in Thailand. Parasit Vectors.

[10] Reiner RC, Perkins TA, Barker CM, Niu T, Chaves LF, Ellis AM (2012). A systematic review of mathematical models of mosquito-borne pathogen transmission: 1970–2010. J R Soc Interface.

[11] Vontas J, David JP, Nikou D, Hemingway J, Christophides GK, Louis C (2007). Transcriptional analysis of insecticide resistance in *Anopheles stephensi* using cross-species microarray hybridization. Insect Mol Biol.

[12] Gubler DJ (1998). Resurgent vector-borne diseases as a global health problem. Emerg Infect Dis.

[13] Mnzava AP, Knox TB, Temu EA, Trett A, Fornadel C, Hemingway J (2015). Implementation of the global plan for insecticide resistance management in malaria vectors: progress, challenges and the way forward. Malar J.

[14] Ranson H, N’guessan R, Lines J, Moiroux N, Nkuni Z, Corbel V (2011). Pyrethroid resistance in African anopheline mosquitoes: what are the implications for malaria control?. Trends Parasitol.

[15] Hemingway J, Hawkes NJ, McCarroll L, Ranson H (2004). The molecular basis of insecticide resistance in mosquitoes. Insect Biochem Mol Biol.

[16] Brooks GT, Harrison A (1963). Relations between structure, metabolism and toxicity of the ‘Cyclodiene’ insecticides. Nature.

[17] Fine BC, Godin PJ, Thain EM (1963). Penetration of pyrethrin I labelled with Carbon-14 into susceptible and pyrethroid resistant houseflies. Nature..

[18] Wood OR, Hanrahan S, Coetzee M, Koekemoer LL, Brooke BD (2010). Cuticle thickening associated with pyrethroid resistance in the major malaria vector *Anopheles funestus*. Parasit Vectors.

[19] Lilly DG, Latham SL, Webb CE, Doggett SL (2016). Cuticle thickening in a pyrethroid-resistant strain of the common bed bug, *Cimex lectularius* L. (Hemiptera: Cimicidae). PLoS One.

[20] Jacobs CG, Braak N, Lamers GE, Van DZM (2015). Elucidation of the serosal cuticle machinery in the beetle *Tribolium* by RNA sequencing and functional analysis of Knickkopf1, retroactive and Laccase2. Insect Biochem Mol Biol.

[21] Vannini L, Bowen JH, Reed TW, Willis JH (2015). The CPCFC cuticular protein family: anatomical and cuticular locations in *Anopheles gambiae* and distribution throughout Pancrustacea. Insect Biochem Mol Biol.

[22] Vannini L, Reed TW, Willis JH (2014). Temporal and spatial expression of cuticular proteins of *Anopheles gambiae* implicated in insecticide resistance or differentiation of M/S incipient species. Parasit Vectors.

[23] Radó-Trilla N, Albà M (2012). Dissecting the role of low-complexity regions in the evolution of vertebrate proteins. BMC Evol Biol.

[24] Iconomidou VA, Willis JH, Hamodrakas SH (2005). Unique features of the structural model of ‘hard’ cuticle proteins: implications for chitin–protein interactions and cross-linking in cuticle. Insect Biochem Mol Biol.

[25] He N, Botelho JM, RJ MN (2007). Proteomic analysis of cast cuticles from *Anopheles gambiae* by tandem mass spectrometry. Insect Biochem Mol Biol.

[26] Cornman RS, Willis JH (2009). Annotation and analysis of low-complexity protein families of *Anopheles gambiae* that are associated with cuticle. Insect Biochem Mol Biol.

[27] McDougall C, Aguilera F, Degnan BM (2013). Rapid evolution of pearl oyster shell matrix proteins with repetitive, low-complexity domains. J R Soc Interface.

[28] Kumari B, Kumar R, Kumar M (2015). Low complexity and disordered regions of proteins have different structural and amino acid preferences. Mol BioSyst.

[29] Imprasittichai W, Roytrakul S, Sudaratana R (2014). A unique insertion of low complexity amino acid sequence underlies protein-protein interaction in human malaria parasite orotate phosphoribosyltransferase and orotidine 5′-monophosphate decarboxylase. Asian Pac J Trop Med.

[30] Fang FJ, Wang WJ, Zhang DH, Lv Y, Zhou D, Ma L (2015). The cuticle proteins: a putative role for deltamethrin resistance in *Culex pipiens pallens*. Parasitol Res.

[31] Zhou D, Hao SH, Sun Y, Chen L, Xiong CR, Ma L (2012). Cloning and characterization of prophenoloxidase A3 (proPOA3) from *Culex pipiens pallens*. Comp Biochem Physiol B Biochem Mol Biol..

[32] Chen L, Zhong DB, Zhang DH, Shi LN, Zhou GF, Gong MQ (2010). Molecular ecology of pyrethroid knockdown resistance in *Culex pipiens pallens* mosquitoes. PLoS One.

[33] Marcombe S, Poupardin R, Darriet F, Reynaud S, Bonnet J, Strode C (2009). Exploring the molecular basis of insecticide resistance in the dengue vector Aedes Aegypti: a case study in Martinique Island (French West Indies). BMC Genomics.

[34] Lv Y, Wang WJ, Hong SC, Lei Z, Fang F, Guo Q (2016). Comparative transcriptome analyses of deltamethrin-susceptible and -resistant *Culex pipiens pallens* by RNA-seq. Mol Gen Genomics.

[35] Ma K, Li X, Hu H, Zhou D, Sun Y, Ma L (2017). Pyrethroid-resistance is modulated by miR-92a by targeting CpCPR4 in *Culex pipiens pallens*. Comp Biochem Physiol B Biochem Mol Biol.

[36] Noh MY, Muthukrishnan S, Kramer KJ, Arakane Y (2015). *Tribolium castaneum* RR-1 cuticular protein TcCPR4 is required for formation of pore canals in rigid cuticle. PLoS Genet.

[37] Noh MY, Kramer KJ, Muthukrishnan S, Kanost MR, Beeman RW, Arakane Y (2014). Two major cuticular proteins are required for assembly of horizontal laminae and vertical pore canals in rigid cuticle of *Tribolium castaneum*. Insect Biochem Mol Biol.

[38] Wang WJ, Lv Y, Fang FJ, Hong SC, Guo Q, Hu SL (2015). Identification of proteins associated with pyrethroid resistance by iTRAQ-based quantitative proteomic analysis in *Culex pipiens pallens*. Parasit Vectors.

[39] Brogdon W, Chan T (2010). Guideline for evaluating insecticide resistance in vectors using the CDC bottle bioassay.

[40] Togawa T, Dunn WA, Emmons AC, Willis JH (2007). CPF and CPFL, two related gene families encoding cuticular proteins of *Anopheles gambiae* and other insects. Insect Biochem Mol Biol.

[41] Mun S, Noh MY, Dittmer NT, Muthukrishnan S, Kramer KJ, Kanost MR (2015). Cuticular protein with a low complexity sequence becomes cross-linked during insect cuticle sclerotization and is required for the adult molt. Sci Rep.

[42] Nkya TE, Poupardin R, Laporte F, Akhouayri I, Mosha F, Magesa S (2014). Impact of agriculture on the selection of insecticide resistance in the malaria vector *Anopheles gambiae*: a multigenerational study in controlled conditions. Parasit Vectors.

[43] Bonizzoni M, Ochomo E, Dunn WA, Britton M, Afrane Y, Zhou G (2015). RNA-seq analyses of changes in the *Anopheles gambiae* transcriptome associated with resistance to pyrethroids in Kenya: identification of candidate-resistance genes and candidate-resistance SNPs. Parasit Vectors.

[44] Pedrini N, Mijailovsky SJ, Girotti JR, Stariolo R, Cardozo RM, Gentile A, Juarez MP (2009). Control of pyrethroid-resistant Chagas disease vectors with entomopathogenic fungi. PLoS Neglect Trop D.

[45] Balabanidou V, Kampouraki A, MacLean M, Blomquist GJ, Tittiger C, Juárez MP, et al. Cytochrome P450 associated with insecticide resistance catalyzes cuticular hydrocarbon production in *Anopheles gambiae*. Proc Natl Acad Sci USA. 2016;113:9268–73. 10.1073/pnas.1608295113 PMC4995928 27439866

[46] Vannini L, Willis JH (2016). Immunolocalization of cuticular proteins in Johnston's organ and the corneal lens of *Anopheles gambiae*. Arthropod Struct Dev..

[47] Andersen SO (2010). Insect cuticular sclerotization: a review. Insect Biochem Mol Biol.

[48] Oussian B, Seifarth C, Müller U, Berger J, Schwarz H. Cuticle differentiation during *Drosophila *embryogenesis. Arthropod Struct Dev. 2006;35:137–52. 10.1016/j.asd.2006.05.003 18089066

