# Supplementary material for: Culex pipiens pallens cuticular protein CPLCG5 participates in pyrethroid resistance by forming a rigid matrix
Source: Parasit Vectors. 2018 Jan 4;11:6. doi: 10.1186/s13071-017-2567-9 (PMC5753453; doi:10.1186/s13071-017-2567-9)
Supplement: Supplementary file 1 — List of primers used for qRT-PCR. (DOC 15 kb) [file 13071_2017_2567_MOESM1_ESM.doc]

Table S1: Primers used in this study

| Purpose | Direction | Primer sequence (5'-3') |
| --- | --- | --- |
| Real-time PCR for CPLCG5 | F | CACCATGCCGGAGTTGTTC |
| Real-time PCR for CPLCG5 | R | GGTTCAGTTGCTTCTGGGAC |
| siRNA for CPLCG5 | F | CCAUUCUUGCCGUUGUCCUTT |
| siRNA for CPLCG5 | R | AGGACAACGGCAAGAAUGGTT |
| Actin | F | AGCGTGAACTGACGGCTCTTG |
| Actin | R | ACTCGTCGTACTCCTGCTTGG |

*F = forward; R = reverse
