# Supplementary material for: Culex pipiens pallens cuticular protein CPLCG5 participates in pyrethroid resistance by forming a rigid matrix
Source: Parasit Vectors. 2018 Jan 4;11:6. doi: 10.1186/s13071-017-2567-9 (PMC5753453; doi:10.1186/s13071-017-2567-9)
Supplement: Supplementary file 2 — List of siRNA sequences used for RNA interference. (DOC 20 kb) [file 13071_2017_2567_MOESM2_ESM.doc]

Table 2

| Name | | Epitope | Length | Amino acid region | Terminus |
| --- | --- | --- | --- | --- | --- |
| Peptide | | NH2-CGATSVTATRG-CONH2 | 11 | 94-103 | C-Term |
| Sequence | MKVAILAVVLALAVASEASWPAGPWGATVVQANNPWPAAHWAGAPWGGAWPGAAWAGAYPYAGAAHWAGAYPYAGAHWGAPAASVAHHAGVVPGATSVTATRGAVHVAPLAGHAVSQKQLNLAPAPGTI | | | | |
