# Supplementary figures and images for: Culex pipiens pallens cuticular protein CPLCG5 participates in pyrethroid resistance by forming a rigid matrix
Source: Parasit Vectors. 2018 Jan 4;11:6. doi: 10.1186/s13071-017-2567-9 (PMC5753453; doi:10.1186/s13071-017-2567-9)

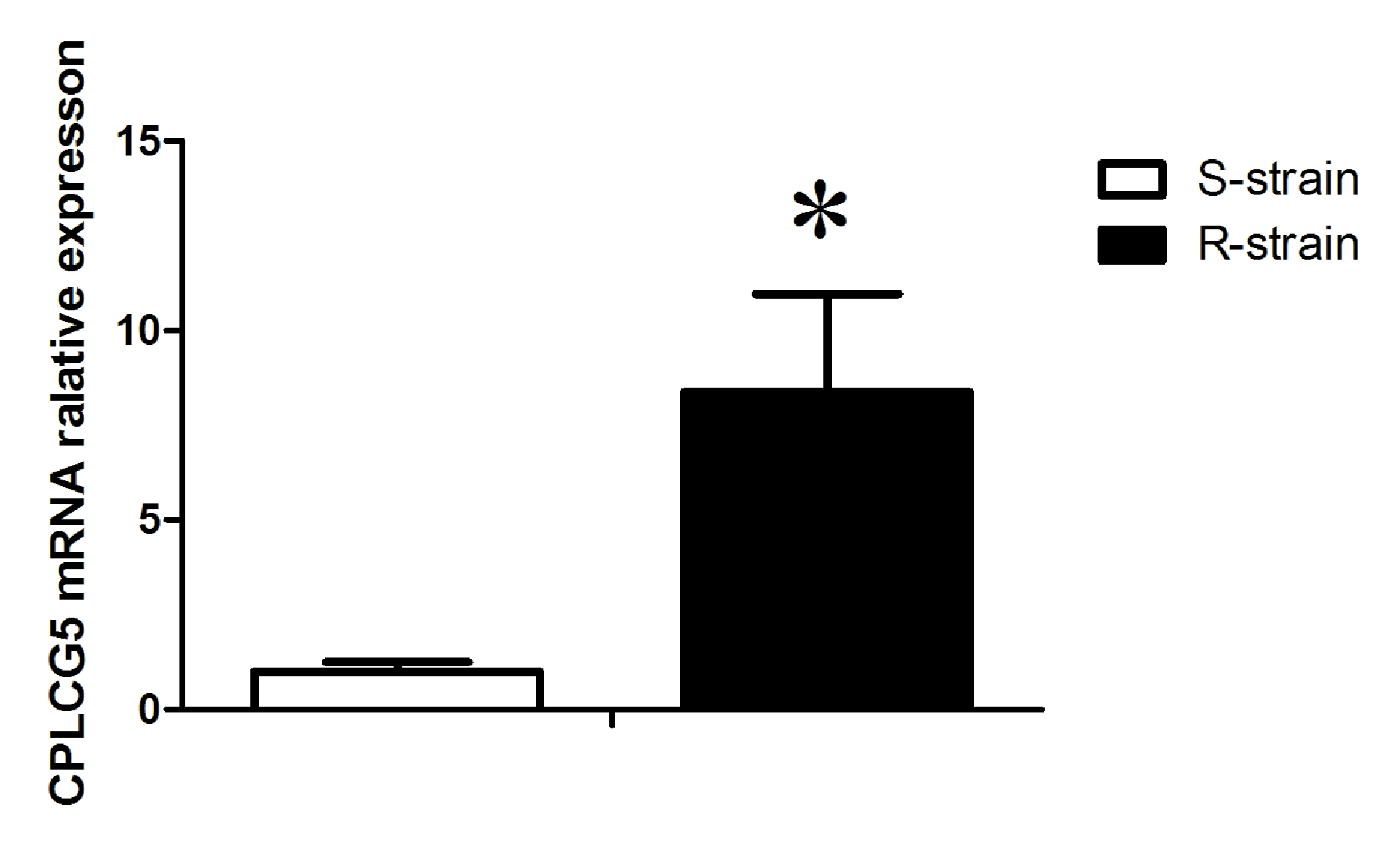

Supplement: Supplementary file 3 — qRT-PCR comparison of the expression of CPLCG5 in DS and DR C. pipiens pallens strains. (PNG 39 kb) [file 13071_2017_2567_MOESM3_ESM.png]

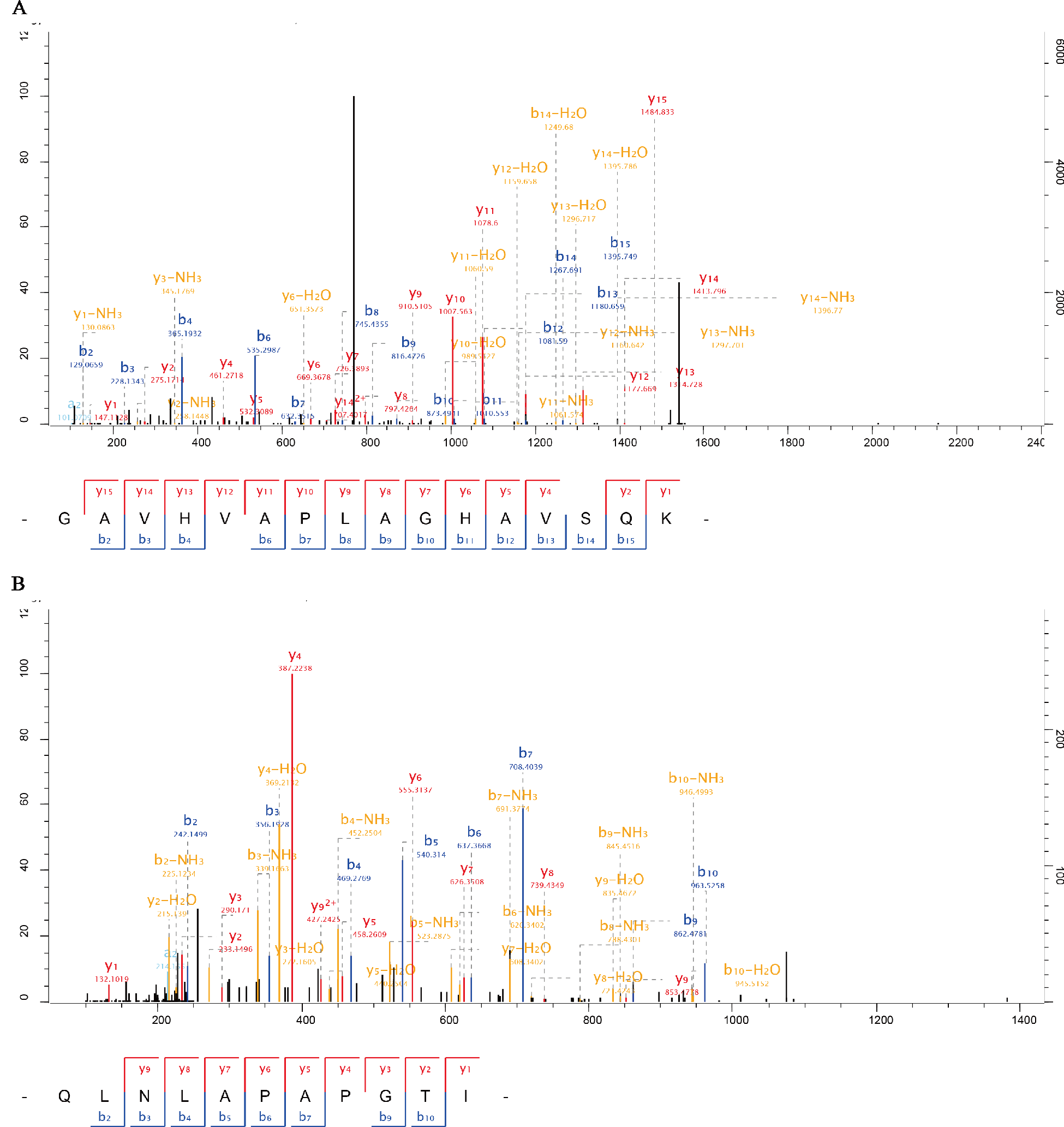

Supplement: Supplementary file 4 — Representative LC-MS/MS spectrum showing a peptide from the CPLCG5 protein. (PNG 184 kb) [file 13071_2017_2567_MOESM4_ESM.png]
