# Supplementary material for: Culex pipiens pallens cuticular protein CPLCG5 participates in pyrethroid resistance by forming a rigid matrix
Source: Parasit Vectors. 2018 Jan 4;11:6. doi: 10.1186/s13071-017-2567-9 (PMC5753453; doi:10.1186/s13071-017-2567-9)
Supplement: Supplementary file 5 — Chi-square tests. (DOCX 19 kb) [file 13071_2017_2567_MOESM5_ESM.docx]

Additional file 5: Chi-square test.

| χ2 | df | P |
| --- | --- | --- |
| 3.399 | 1 | >0.05 |
| 5.385 | 1 | < 0.05 |
| 4.204 | 1 | < 0.05 |
| 5.908 | 1 | < 0.05 |
| 4.326 | 1 | < 0.05 |
| 3.941 | 1 | < 0.05 |
| 9.907 | 1 | < 0.01 |
| 15.927 | 1 | < 0.0001 |
